# MicroRNA-3148 modulates differential gene expression of the SLE-associated *TLR7 *variant

**DOI:** 10.1186/ar3939

**Published:** 2012-09-27

**Authors:** Y Deng, J Zhao, D Sakurai, KM Kaufman, JC Edberg, RP Kimberly, DL Kamen, GS Gilkeson, CO Jacob, RH Scofield, CD Langefeld, JA Kelly, ME Alarcón-Riquelme, JB Harley, TJ Vyse, BI Freedman, PM Gaffney, KM Sivils, JA James, TB Niewold, RM Cantor, W Chen, BH Hahn, EE Brown, BP Tsao

**Affiliations:** 1 University of California, Los Angeles, CA, USA; 2 Center for Autoimmune Genomics & Etiology, Cincinnati Children's Hospital Medical Center, Cincinnati, OH, USA; 3 US Department of Veterans Affairs Medical Center, Cincinnati, OH, USA; 4 University of Alabama at Birmingham, Birmingham, AL, USA; 5 Medical University of South Carolina, Charleston, SC, USA; 6 Keck School of Medicine, University of Southern California, Los Angeles, CA, USA; 7 Arthritis & Clinical Immunology Program, Oklahoma Medical Research Foundation, Oklahoma City, OK, USA; 8 University of Oklahoma Health Sciences Center, Oklahoma City, OK, USA; 9 US Department of Veterans Affairs Medical Center, Oklahoma City, OK, USA; 10 Wake Forest University Health Sciences, Wake Forest, NC, USA; 11 Centro de Genómica e Investigación Oncológica (GENYO), Pfizer-Universidad de Granada-Junta de Andalucia, Granada, Spain; 12 King's College London, UK; 13 Wake Forest School of Medicine, Winston-Salem, NC, USA; 14 Gwen Knapp Center for Lupus and Immunology Research, University of Chicago, IL, USA

## Background

We identified the G allele of *TLR7 *3'-UTR SNP (rs3853839) associated with increased *TLR7 *transcripts, a more pronounced IFN signature and risk for SLE in 9,274 Eastern Asians (*P*_combined _= 6.5 × 10^-10^) [1]. The current study sought replication of SLE-associated SNP(s) in non-Asian ancestries and explored molecular mechanisms underlying an identified gene variant that affects TLR7 expression.

## Methods

We conducted genotyping, imputation and association for 98 to 116 SNPs (varying among different ancestries) covering 80 kb of *TLR7-TLR8 *in European Americans (EA), African Americans (AA) and Hispanics enriched for the Amerindian-European admixture (HS). Haplotype-based conditional testing was conducted to distinguish independent association signals. Mantel-Haenszel testing was used in transancestral meta-analysis. Association of genotypes with TLR7 expression was examined using RT-PCR, flow cytometry and reporter assays. Pyrosequencing was used to measure allelic variations in *TLR7 *transcript levels.

## Results

The rs3853839 was confirmed as the only variant within *TLR7-TLR8 *exhibiting consistent and independent association with SLE in our transancestral fine-mapping (*P*_meta _= 7.5 × 10^-11^, OR (95% CI) = 1.24 (1.18 to 1.34)) in 13,339 subjects of EA (3,936 cases vs. 3,491 controls), AA (1,679 vs. 1,934) and HS (1,492 vs. 807) ancestries. PBMCs from normal G-allele carriers exhibited elevated levels of *TLR7 *mRNA (*P *= 0.01 in men and *P *= 0.02 in women) and protein (*P *= 0.009 in men and *P *= 0.038 in women). PBMCs from heterozygotes exhibited higher G/C allele ratios of *TLR7 *transcripts 4 hours after incubation with actinomycin D (inhibitor of transcription initiation) (*P *= 0.04), indicating slower degradation of G allele-containing transcript. The nonrisk allele, but not the risk allele, was predicted to match microRNA-3148 (miR-3148) at the second base in the binding site. Transcript levels of miR-3148 and TLR7 were inversely correlated in PBMCs from 16 SLE patients and 21 controls (*R*^2 ^= 0.255, *P *= 0.001), suggesting miR-3148 modulating TLR7 expression. Overexpression of miR-3148 via transfection into HEK 293 cells led to a more than twofold reduction in luciferase activity driven by the *TLR7 *3'-UTR segment containing the nonrisk allele than that containing the risk allele (*P *= 0.001).

## Conclusion

We identified and confirmed a genome-wide significant association between rs3853839 and SLE susceptibility in 22,613 subjects of Eastern Asian, EA, AA and HS ancestries (*P*_meta _= 6.4 × 10^-19^, OR (95% CI) = 1.26 (1.20 to 1.32)). Reduced modulation by miR-3148 confers slower degradation of the risk allele containing *TLR7 *transcript, resulting in elevated levels of gene products and a more robust type I IFN signature.
